# Cellular response of keratinocytes to the entry and accumulation of nanoplastic particles

**DOI:** 10.1186/s12989-024-00583-9

**Published:** 2024-04-29

**Authors:** Leisha Martin, Kayla Simpson, Molly Brzezinski, John Watt, Wei Xu

**Affiliations:** 1 Department of Life Sciences, College of Science, Texas A&M University - Corpus Christi, 6300 Ocean Dr, 78412 Corpus Christi, TX USA; 2 Center for Integrated Nanotechnologies, Los Alamos National Laboratory, Albuquerque, NM USA

**Keywords:** Nanoplastics, Skin barrier, Transepidermal transport, Keratinocyte, Dermal fibroblast, Inflammation

## Abstract

Plastic accumulation in the environment is rapidly increasing, and nanoplastics (NP), byproducts of environmental weathering of bulk plastic waste, pose a significant public health risk. Particles may enter the human body through many possible routes such as ingestion, inhalation, and skin absorption. However, studies on NP penetration and accumulation in human skin are limited. Loss or reduction of the keratinized skin barrier may enhance the skin penetration of NPs. The present study investigated the entry of NPs into a human skin system modeling skin with compromised barrier functions and cellular responses to the intracellular accumulations of NPs. Two in vitro models were employed to simulate human skin lacking keratinized barriers. The first model was an ex vivo human skin culture with the keratinized dermal layer (stratum corneum) removed. The second model was a 3D keratinocyte/dermal fibroblast cell co-culture model with stratified keratinocytes on the top and a monolayer of skin fibroblast cells co-cultured at the bottom. The penetration and accumulation of the NPs in different cell types were observed using fluorescent microscopy, confocal microscopy, and cryogenic electron microscopy (cryo-EM). The cellular responses of keratinocytes and dermal fibroblast cells to stress induced by NPs stress were measured. The genetic regulatory pathway of keratinocytes to the intracellular NPs was identified using transcript analyses and KEGG pathway analysis. The cellular uptake of NPs by skin cells was confirmed by imaging analyses. Transepidermal transport and penetration of NPs through the skin epidermis were observed. According to the gene expression and pathway analyses, an IL-17 signaling pathway was identified as the trigger for cellular responses to internal NP accumulation in the keratinocytes. The transepidermal NPs were also found in co-cultured dermal fibroblast cells and resulted in a large-scale transition from fibroblast cells to myofibroblast cells with enhanced production of α-smooth muscle actin and pro-Collagen Ia. The upregulation of inflammatory factors and cell activation may result in skin inflammation and ultimately trigger immune responses.

**Supplementary Information:**

The online version contains supplementary material available at 10.1186/s12989-024-00583-9.

## Background

Environmental pollution from overproduction, transportation accidents resulting in the accidental release, inadequate waste handling and disposal procedures coupled with ill-fated recycling programs are resulting in significant, widespread plastic pollution. The properties of plastic that once made it attractive for use, such as durability, resistance to wear, and low cost of production have become problematic as discarded plastics persists in the environment indefinitely while manufacturing of new plastics continues to increase. Over time, environmental conditions and photodegradation can cause bulk plastic waste to be broken down into nanoscale fragments, nanoplastics (NPs) [1–3]. In addition to the degradation of bulk waste, nano and microscale plastic particles, may also enter the environment from industrial waste and personal care products. Many commercial personal care products contain microbeads, and recently, NPs have been found in these products [4]. These small plastic fragments are stable and durable and may persist in the environment for hundreds to thousands of years [5].

Humans are inevitably exposed to plastic particles on many occasions through different exposure routes. Nanoplastic exposure can occur via ingestion, inhalation, or absorption through the skin [6–8]. Due to their small size, NPs have a large surface area and high surface energy and have the potential for deep biological and vital organ penetration [9, 10]. Compared to other organs, human skin employs a unique structure to provide protection against external stressors. Recent studies suggested the ubiquitousness of the micro and nanoplastics in the environment even in some extreme conditions, such as the North Atlantic Ocean and deep oceans [11, 12]. As the first barrier against pathogens, chemicals, and particles from the environment, human skin is constantly exposed to environmental plastic particles. The keratinized layers on the surface of the skin help prevent environmental particles from entering the epidermis. However, keratinized layers are often missing in areas of injured or diseased skin. External factors, such as detergents, solvents, and excessive hydration, can all remove the stratum corneum and the underlying lipid bilayers. Internal factors from the hosts that can compromise the skin barrier include age [13], inflammatory skin diseases [14, 15], and skin allergies [16, 17]. Loss of the protective barrier makes human skin permeable to many particles and chemicals in the environment [18]. Therefore, patients with damaged skin barriers are at higher risk of NP absorption through epidermal transport.

Inside the skin, NPs can be taken up by epidermal keratinocytes and ultimately to reach dermal cells through transepidermal transportation. The NPs retained in the epidermis may promote inflammation by triggering the production of proinflammatory cytokines [19, 20]. In addition, the environmental NPs often accumulate various organic compounds on their surfaces which may contribute to triggering more severe inflammatory reactions in the skin [21]. However, previous studies on NP dermatoxicity primarily used skin cells as models, which failed to represent the characteristics of real human skin. In this study, besides monitoring the uptake of NPs by skin keratinocytes and dermal fibroblast cells using fluorescent imaging and cryo-EM, two human skin equivalents, ex vivo skin culture and 3D keratinocyte/fibroblast cell co-culture, were used to demonstrate the transportation of NPs through the skin epidermis without the protection of stratum corneum. The accumulation of NPs in skin cells demonstrated the impacts on cell genetic response and cellular behaviors. The results of the study may provide useful information contributing to a better understanding of the potential risk of human skin exposure to environmental NPs, particularly in the population with compromised barrier functions in skin.

## Results

### NP uptake was found in both keratinocytes and dermal fibroblast cells

**Fig. 1:**
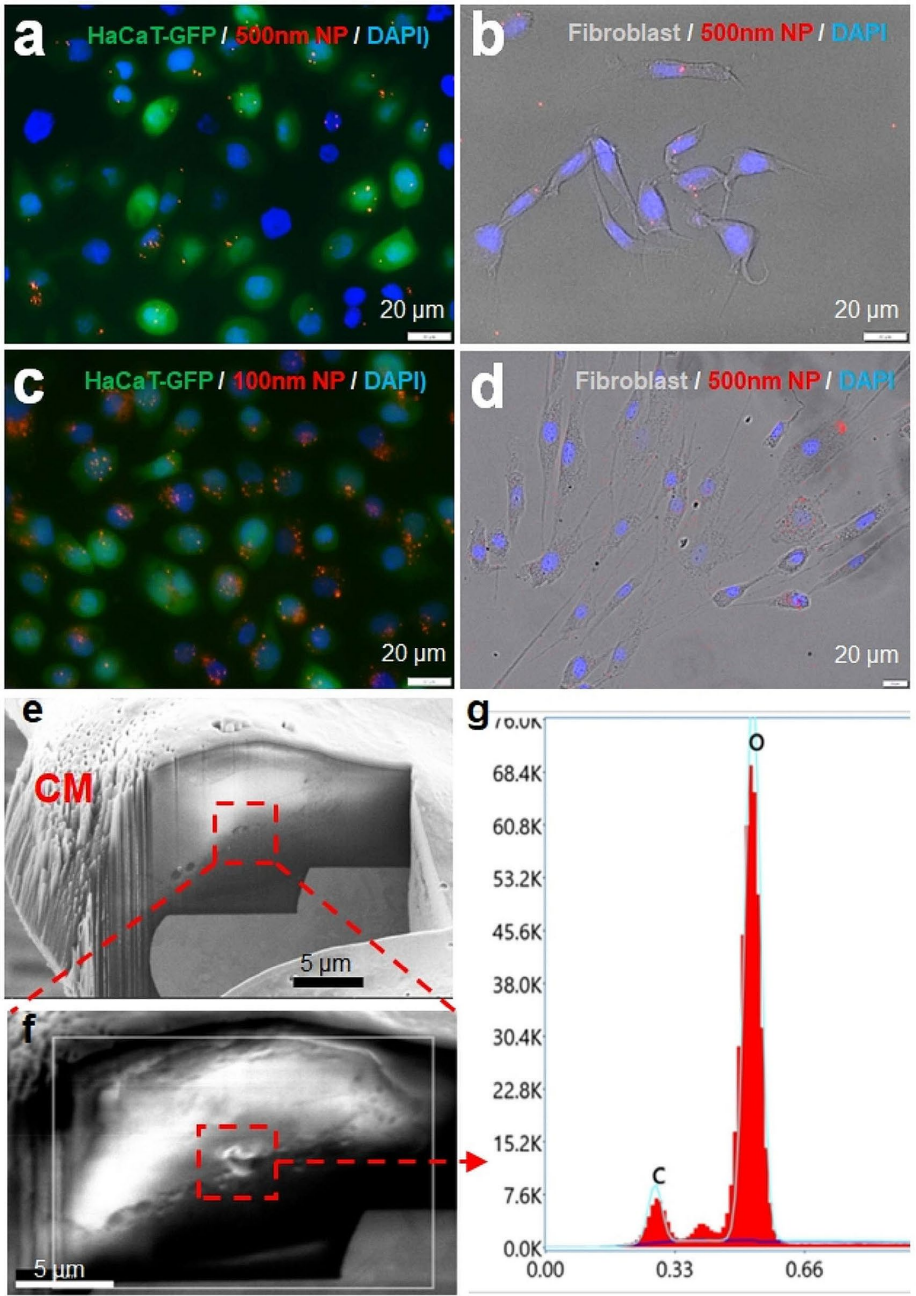
Accumulation of NPs in keratinocytes and dermal fibroblasts. 500 nm polystyrene NPs were able to enter keratinocytes (**a**) and dermal fibroblasts (**b**). 100 nm polystyrene NPs also accumulated in keratinocytes (**c**) and fibroblasts (**d**) The cryo-FIB image showed the possible location of intracellular NPs (**a**, scale bar: 20 μm). The particles in this location (**b**, scale bar: 5 μm) were identified by the XEDS to be high carbon (**C**) density particles which are different than the intracellular background with high level of oxygen (O) (**c**). CM: cell membrane. Resolution of XEDS is 75 nm x 75 nm

NPs with two different sizes, 100 nm and 500 nm in diameter, were both taken up by the skin keratinocytes and fibroblast cells after 16 h of incubation. It was difficult to visualize the individual NPs inside or on the surface of cells due to their sizes. The fluorescent signal intensities were used to estimate the abundance of the NPs. With regular fluorescence microscopy, co-localization of NPs and cells was observed. Limited NPs with 500 nm diameter were found to co-localize with keratinocytes (Fig. 1a) and dermal fibroblast cells (Fig. 1b). NPs accumulated to form clusters. Those clusters overlapped with about 60% of cells in the field (Fig. 1a and b). In contrast, 100 nm NPs were found to co-localize with more keratinocytes (Fig. 1c) and fibroblast cells (Fig. 1d) compared to 500 nm NPs. Over 90% of the keratinocytes or fibroblast cells overlapped with the 100 nm NP signals. Higher NP densities around nuclei were noted in dermal fibroblast cells as shown in the confocal imaging (Supplementary Data Fig. S1).

To confirm the entry of the NPs to skin cells, cryogenic focused (cryo-EM) was used to visualize the intracellular NPs. A cross-section of the cell was first formed using cryogenic focused ion beam (cryo-FIB) milling and then the exposed face was imaged using cryogenic scanning electron microscopy (cryo-SEM). High contrast particles were observed intracellularly (Fig. 1e, Supplementary Data Fig. S2a), which suggested the inclusion of a high-density particle. This was then further characterized by X-ray energy dispersive spectroscopy (XEDS) mapping (Fig. 1f and g, Supplementary Data Fig. S2b). From XEDS the intracellular NPs showed a strong carbon signal along with an associated reduction in signal from oxygen (Fig. 1g, Supplementary Data Fig. S2c). In contrast, other regions of the cytoplasm showed a strong O signal, co-located with a weak C signal, which is characteristic of the hydrate intercellular region (Fig. 1g, Supplementary Data Fig. S2c). The strong C and weak O signal of the high contrast particles indicate polystyrene nanoparticles (Supplementary Data Fig. S2d).

### NPs penetrate the skin epidermis without the protection of keratinized barriers

**Fig. 2:**
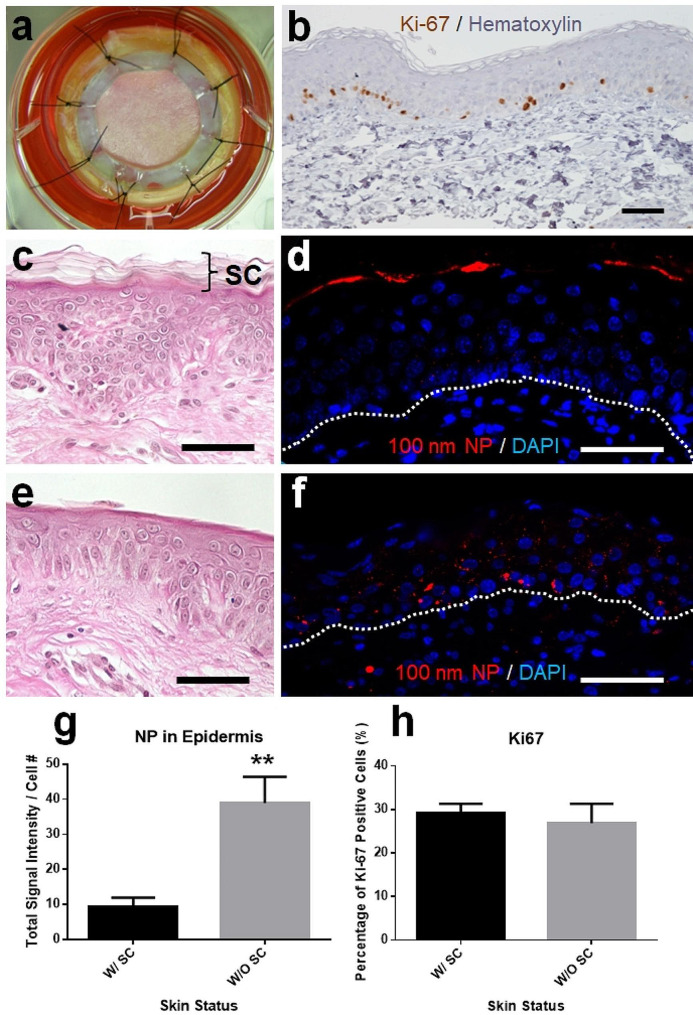
Penetration of NPs into the skin with missing stratum corneum in ex vivo culture. The human skin explant was cultured in vitro (**a**) and the proliferating progenitor keratinocytes were identified at the basal area of the epidermis after 4 days of in vitro culture (**b**, cells with red stain on nuclei). With the highly keratinized stratum corneum layer of the skin (**c**), most NPs were accumulated at the stratum corneum layer (**d**). In the skin culture without stratum corneum (**e**), NPs were found in all layers of the epidermis (**f**). The intra-epidermal NPs were quantified by measuring the signal intensities (g, y-axis unit: pixels/cell) and the proliferation rates of basal epidermal cells (**h**) were calculated in all samples. SC: stratum corneum. Dashed line: basement membrane. Scale bar: 50 μm

The penetration of NPs through the human skin epidermis was simulated using a skin ex vivo culture model (Fig. 2a). The skin culture maintained the natural status of the real skin for at least four days since many basal layer keratinocytes were under proliferating as shown by the presence of Ki-67 positive nuclei (Fig. 2b, nuclei with red color). The healthy skin under normal conditions has a thick layer of highly keratinized stratum corneum layer on top of the keratinocytes (labeled S in Fig. 2c). This stratum corneum layer sufficiently kept the 100 nm NPs from skin penetration as most of the NP signals were seen on top of the skin after 48 h of incubation (Fig. 2d). The stratum corneum can be absent in many skin disorders, this situation was simulated by the physical removal of the stratum corneum (Fig. 2e). Without the protection of stratum corneum, the skin epidermis became more permeable to NPs as a significantly increased number of NPs were seen in all layers of epidermis (Fig. 2f). The average NP signal intensity per cell in the skin epidermis without stratum corneum was 38.87 ± 7.52 pixels/cell while it was 9.23 ± 2.70 pixels/cell in the skin with the stratum corneum (*p* = 0.01, Fig. 2g).

Although more NPs were found in the epidermis of stratum corneum missing skin culture, there was no significant difference in the proliferation rates of the basal layer keratinocytes. The percentages of proliferating basal keratinocytes in control and stratum corneum missing skin cultures were 29.15 ± 2.18% and 26.90 ± 4.42%, respectively (Fig. 2h).

### Accumulation of NPs in keratinocytes may induce inflammation and tissue remodeling

**Fig. 3:**
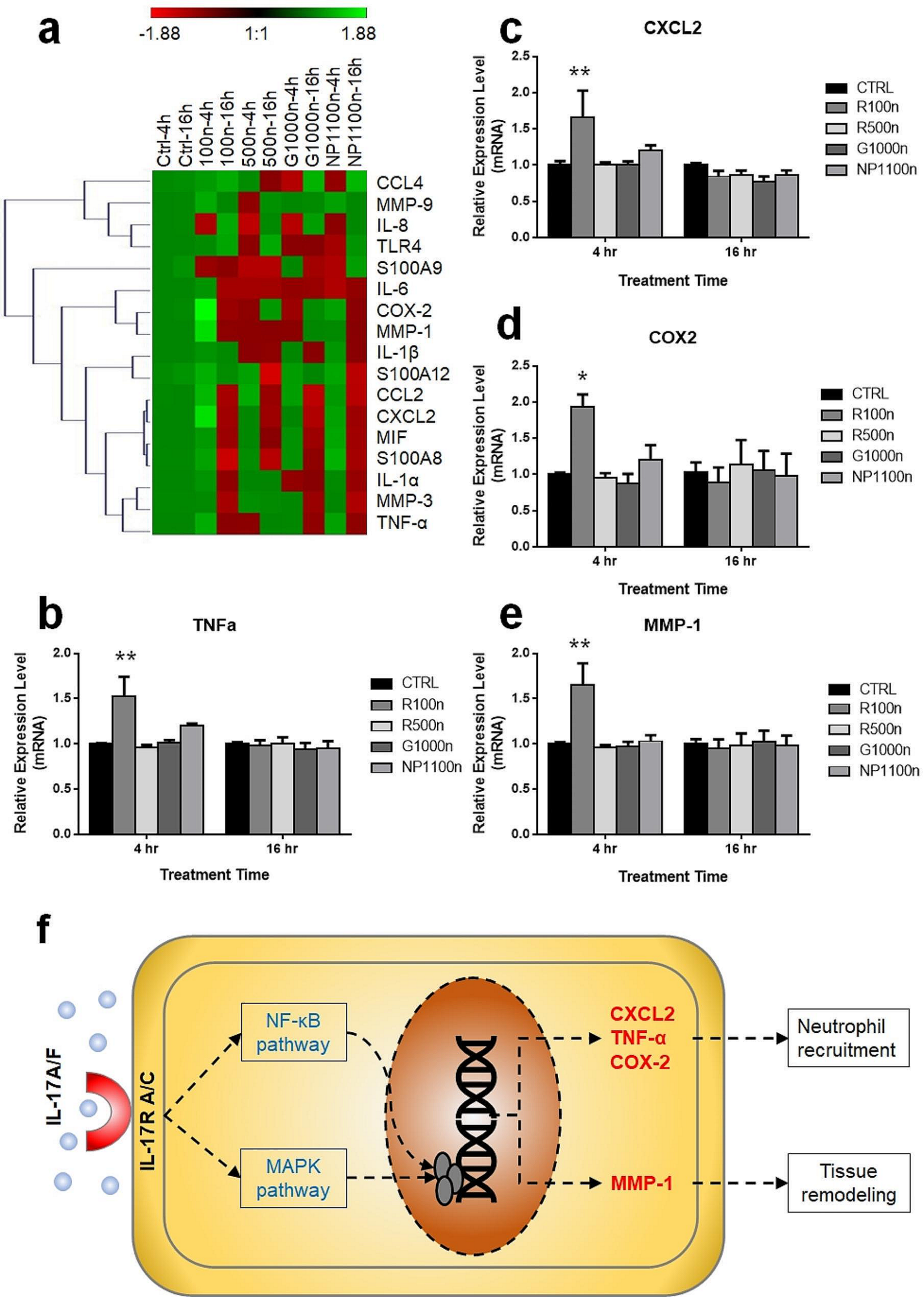
Genetic response of keratinocytes to the stress of intracellular NPs. The overall gene expression patterns under different NP stress were demonstrated by the heatmap with the relative mRNA levels by qPCR (**a**). The expression levels of *tnfa* (**b**), *cxcl2* (**c**), *ptgs2* (COX2 encoding gene, **d**), and *mmp1* (**e**) were found to be upregulated by 100 nm NPs. All these genes were involved in the IL17/IL17R regulatory pathway (f, constructed based on the KEGG PATHWAY: hsa04657

Accumulation of NPs in keratinocytes altered the expression levels of several inflammation-related genes. NPs in both 100 nm and 500 nm diameters were tested. The applications of G1000n and NP1100n particles to keratinocytes were to identify the potential effects of the fluorescent tags on the gene expression of keratinocytes. The results showed no significant difference in the expression of all 17 tested genes when the cells were exposed to the fluorescent G1000n and non-tagged NP1100n (Fig. 3 and Supplementary Data Fig. S4). Amount all 17 genes tested in this study (Fig. 3a), four genes showed altered expression patterns in response to the stimulation of 100 nm NPs within four hours. The expression level of two cytokines, *tnfa*, and *cxcl2*, reached 1.52 ± 0.22 fold (*p* = 0.0011) and 1.66 ± 0.37 fold (*p* = 0.0095) respectively, compared to naïve cells (Fig. 3b). The other two genes, *ptgs2* (COX2 encoding gene) and *mmp1*, demonstrated similar upregulation in their expression patterns at four-hour post stimulation of 100 nm NPs. Their expression levels were enhanced to 1.93 ± 0.18-fold (*p* = 0.0300, Fig. 3c) and 1.65 ± 0.25-fold (*p* = 0.0026, Fig. 3d), respectively. The four upregulated genes were annotated to the genetic regulatory pathways in the KEGG database and a human IL17 regulatory pathway (hsa04657) was identified. Within the IL17 family, IL17A and IL17F were found to be the upstream regulators to produce CXCL2, TNF-α, COX-2, and MMP-1 in keratinocytes. The expression patterns of all other 13 tested genes under the stress of NPs were included in Supplementary Data Fig. S3. Despite the alterations in gene expression, there was no significant change in the proliferation of cells as indicated in Fig. 2h. In addition, no alterations in cell migration in response to NP stimulation were identified (Supplementary Data Fig. S4).

### NPs progressively penetrated the 3D keratinocyte culture in vitro

**Fig. 4:**
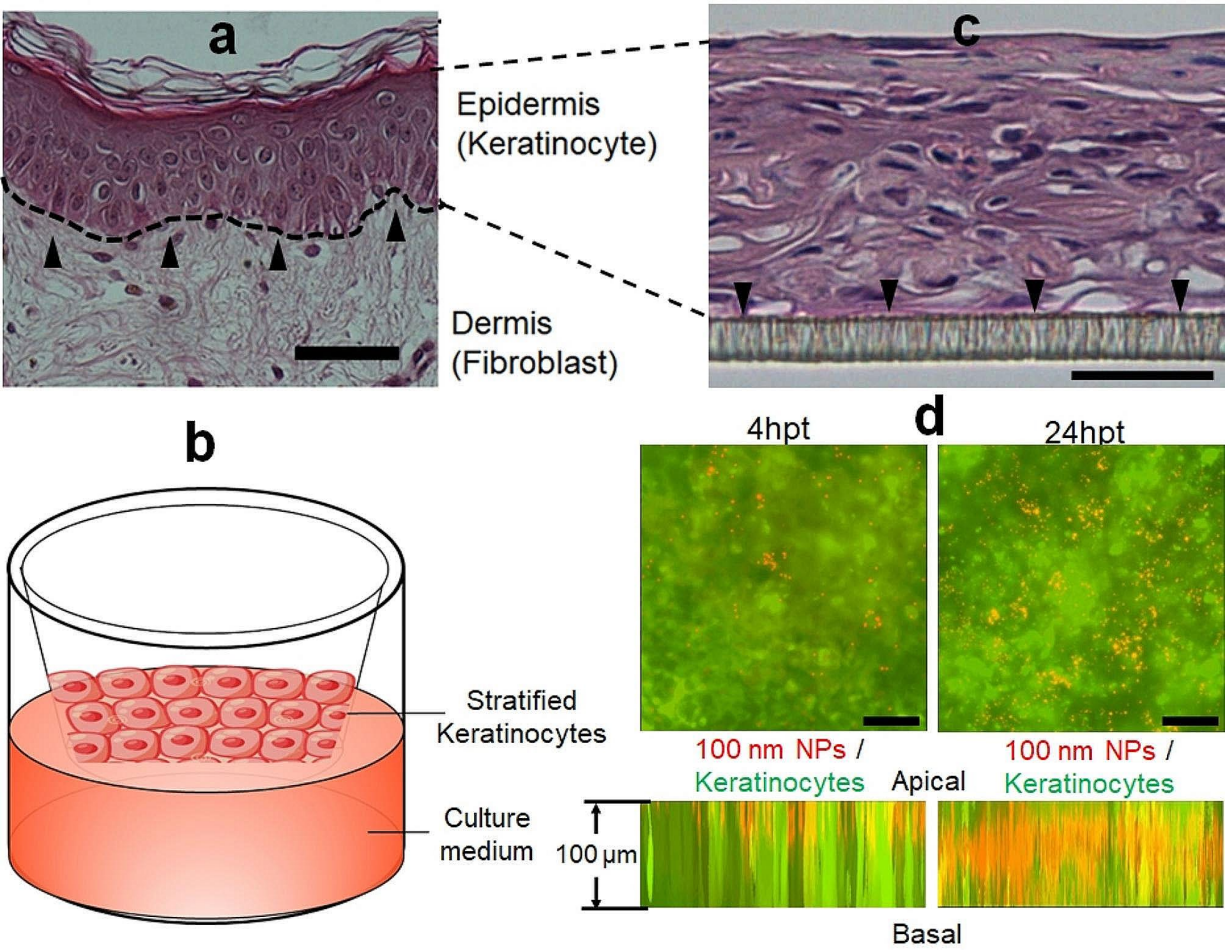
Transepidermal penetration of NPs. The epidermal structure of human skin (**a**) was simulated by the 3D keratinocyte culture (**b**) which showed well-stratified keratinocytes as seen in real human skin (**c**). The depth of NPs in the epidermis and densities of NPs at the basal layers of the epidermis at 4 h post-treatment (hpt; left) and 16 hpt (right) were visualized by in-depth 3D scanning of the culture constructs (**d**). Scale bar: 50 μm

To assess the penetration of NPs through the skin epidermis, an in vitro keratinocyte 3D culture model was built. Since the keratinocytes in normal human skin comprise multiple layers (Fig. 4a), the keratinocytes in the cell culture inserts were stratified and cultured at the air-liquid interface (Fig. 4b). Stratification resulted in the generation of multiple keratinocyte layers over the membrane of the insert (Fig. 4c) while the stratum corneum layer was not developed (Fig. 4a and c). The penetration of 100 nm NPs occurred progressively along the time course. After 4 h of penetration, very few NPs were seen at the basal layer of the cell culture (Fig. 4d, top left). In contrast, many more NPs were found at the basal layer after 24 h of penetration (Fig. 4d, top right). The side view of the stratified keratinocyte culture confirmed the finding. The NPs originally applied to the surface of the cell culture only penetrated the superficial layers of keratinocytes after 4 h of incubation (Fig. 4d, bottom left) while the majority of the NPs reached the layers of keratinocytes in the middle of the construct (Fig. 4d, bottom right).

### Transepidermal NPs reached and activated the co-cultured dermal fibroblast cells

**Fig. 5:**
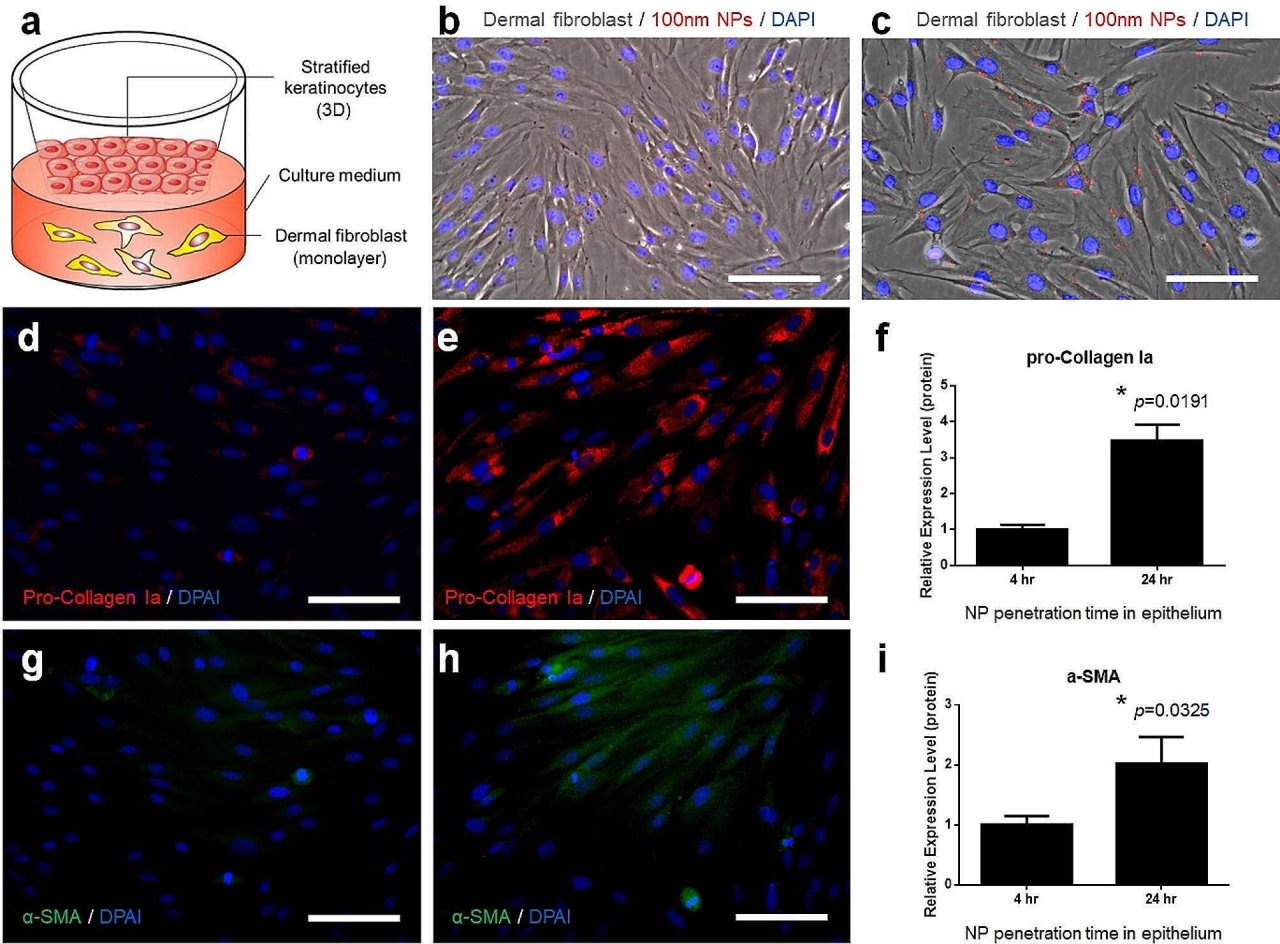
Effects of NPs on co-cultured dermal fibroblasts. The NPs released by the epidermis through the pores on the cell culture inserts were taken up by dermal fibroblasts (**a**) at 4 h (**b**) and 24 h (**c**) after epidermal treatment. The pro-collagen Ia levels in fibroblasts at 4 h (**d**) and 16 h (**e**) were estimated by immunofluorescence. The α-SMA levels in fibroblast cells were also measured after 4 h (**g**) and 24 h (**h**) NP treatment on co-cultured 3D keratinocyte cultures. The quantification of α-SMA levels was achieved by immunofluorescence as well (**i**). Scale bar: 50 μm

The impacts of transepidermal NPs on the stromal cells in the dermis of human skin were assessed using the 3D keratinocyte/fibroblast cell co-culture model. The 3D keratinocyte culture constructs containing inserts were hung over the monolayer dermal fibroblast cells grown in the wells (Fig. 5a). The distance between the basal layer of the 3D keratinocyte culture and the fibroblast cells was 0.9 mm according to the manufacturer’s instructions. The membrane of the cell culture insert served as the basement membrane which allows the attachment of basal layer keratinocytes. The pore size of the insert membrane is 0.4 μm which is relevant to the size of the pores on the basement membrane (0.79 μm on average) [22]. In addition, the pores on the insert membrane allow the 100 nm NPs to traverse the membrane and interact with the co-cultured dermal fibroblast cells. With the different duration of NP transepidermal transportation, the number of NPs transported across the membrane differed. This was demonstrated by the numbers of NPs accumulated in the co-cultured fibroblast cells shown in Fig. 5b and c. Only a small number of NPs was found in the fibroblast cells after 4 h of epidermal penetration (Fig. 5b). In contrast, NP signals were detected in all cells in co-cultured fibroblast cells after 24-hour transepidermal transportation (Fig. 5c). As a control, 1.0 ppm NPs with 100 nm (Supplementary Data Fig. S5a) or 500 nm (Supplementary Data Fig. S5b) were directly applied to the top of the cell culture inserts with the dermal fibroblast cells co-cultured underneath. With a 24-hour incubation, a great number of 100 nm NPs were taken by the fibroblast cells while no signals for 500 nm NPs were detected in co-cultured fibroblast cells. This suggested the sufficient blockage of 500 nm NP penetration by the cell culture insert due to the pore size (0.4 μm).

Subsequently, the differing numbers of transepidermal NPs altered the status of the co-cultured dermal fibroblast cells. Low levels of pro-Collagen Ia (pro-Col Ia) were found in the fibroblast cells cultured under the keratinocytes with 4-hour NP penetration (Fig. 5d). In contrast, 24-hour NP penetration increased the production of pro-Col Ia in fibroblast cells (Fig. 5e) to 3.47 ± 0.45-fold (*p* = 0.0191, Fig. 5f). Similarly, a significant elevation of α smooth muscle actin (α-SMA) level in the co-cultured fibroblast cells was observed when increasing the NP penetration time from 4 h (Fig. 5g) to 24 h (Fig. 5h). A 2.02 ± 0.44-fold change was identified with the quantification analysis (Fig. 5i).

## Discussion

The motivation of the present study was to visualize the transportation of NPs through the skin epidermis and identify the cellular responses to the intracellular accumulation of NPs. Therefore, we applied two skin equivalent constructs to demonstrate the NP penetration. The ex vivo skin culture maintained the most natural characteristics of real human skin including the viable keratinocytes and stromal cells in the dermis as well as the protein components in the basement membrane and dermis. It is also a great tool to demonstrate the importance of the stratum corneum as the barrier. Previous studies suggested that the entry of NPs into the epidermis of healthy skin was primarily through hair follicles [23]. However, the skin stratum corneum can be lost by many means, such as sunburn [24]. In our study, the stratum corneum was removed to mimic similar skin conditions. NPs migrated to the different layers of the epidermis with the removal of the stratum corneum. Very few NPs were seen to enter the dermal area of the skin culture. The migration of NPs through the dense irregular connective tissue in the dermis may be difficult without the help of cellular carriers. The depth of particle penetration through the skin may depend on the materials and surface properties of the particles. A previous study on the skin absorption of TiO_2_ and ZnO on the skin with sunburn showed very different migration patterns of these two particles in the skin [24].

The 3D keratinocyte/fibroblast cell co-culture model simplified the skin system by maintaining only two types of cells, which are the dominant cells in the epidermis and dermis respectively. This model allows us to monitor the penetration process of the NPs in the epidermis. The transepithelial transport of nanoparticles has been extensively studied for drug deliveries. Nanoparticles can penetrate the epithelia through transcellular and paracellular transport pathways depending on the types of nanoparticles and epithelia [25–27]. In the skin epidermis, nanoparticles with a 100 nm diameter will be extremely difficult to transport paracellularly because of the large number of cell-cell junctions, especially the tight junctions [28]. Although the locations of NPs in the epidermis could not be identified due to the limitation of the imaging technique, it is reasonable to speculate that the majority of intraepidermal NPs were in the keratinocytes, which caused the cellular behavioral changes through the internal effect. Interestingly, we also noticed that not all the NP particles traveled across the entire layers of the epidermis and reached the dermal areas based on the results from the ex vivo and 3D cell culture models. Although we could not quantify the NPs that penetrated the epidermis, we found a reduced number of NPs accumulated in the co-cultured dermal fibroblast compared to the fibroblast cells incubated with the original concentration of NPs (Fig, 5c vs. Supplementary Data Fig. S5a).

Additionally, the involvement of dermal fibroblast cells in the host response to NPs was also investigated using the co-culture model. Since the sizes of the pores on the keratinocyte culture membrane are comparable with the actual pore sizes on the basement membrane between the epidermis and dermis in the skin, penetration of NPs through the membrane simulated the entry of NPs into the dermal layer in real human skin. As a heterogeneous cell population, the dermal fibroblast cells were believed to be involved in the reconstruction of the dermal extracellular matrix [29], the development of hair follicles [30], as well as epithelial-mesenchymal reactions [31]. The quiescent dermal fibroblast cells can be activated by a number of internal and external factors including skin diseases and disorders [32–34]. The activation of dermal fibroblast cells was also observed in our study. Although the positive correlation between the numbers of NPs in dermal fibroblast cells and the levels of fibroblast cell activation was identified (Fig. 5), it is unclear whether the cell activations were triggered by the NPs directly or through the paracrine regulation by co-cultured keratinocytes which were also treated with NPs. The paracrine regulatory factors secreted by the keratinocytes may also be an important factor for fibroblast activation [35–37].

The factors that were produced by keratinocytes in response to the NP accumulation included TNF-α, CXCL2, COX-2, and MMP-1, which were all downstream factors of the IL-17 pathway according to the result from KEGG pathway analysis. The TNF-α, CXCL2, and COX-2 are well-known factors involved in inflammatory responses. One of the critical roles is to recruit the neutrophils in the skin [38, 39]. These factors may be released to the dermal area for neutrophil attraction and dermal fibroblast cell activation [35, 40, 41]. It is also interesting to see the level of MMP-1 in keratinocytes was upregulated by the NP treatment. Although primarily produced by dermal fibroblast cells, MMP-1 in keratinocytes was reported to be correlated to skin damage, such as UV irradiation [42], diabetic chronic wounds [43], and oxidative stress [44]. Recent studies suggested that oxidative stress is the major cellular response to micro and nanoplastic stimulation in many other types of cells [45–47]. Therefore, it is likely that the upregulation of MMP-1 in keratinocytes was a downstream reaction of the oxidative stress initiated by the intracellular NP accumulation.

Despite the valid experimental models and methods applied to this research, the limitation of this study is the application of commercial nanopolystyrene particles in the experiments. Because of the modification of these NP particles for fluorescently labeling purposes, some surface properties of the NPs were altered. For example, the NPs were modified on the amine, carboxyl, or sulfate groups to tag different types of fluoresceine. While we characterized the surfaces of these NPs with different modifications, amine modification significantly altered the zeta potential of the polystyrene nanoparticles (+ 40 mV) compared to the non-labeled polystyrene beads (-40 mV). In this study, we used the carboxyl-modified NPs which showed a minimal modification in zeta potential (-44.5∼-58 mV). This minimizes the influence of the fluorescent labels on our study. However, the NP particles used in this study cannot fully simulate nanoplastic particles in the environment since the environmental particles are known to be colonized by ‘ecocorona’ containing various environmental compounds [48]. While the non-conditioned NP particles used in this study allowed us to understand skin penetration and cellular accumulation and related mechanisms, our ongoing study focused on the environmentally conditioned NP particles which will provide more environmentally relevant results in the future.

**Fig. 6:**
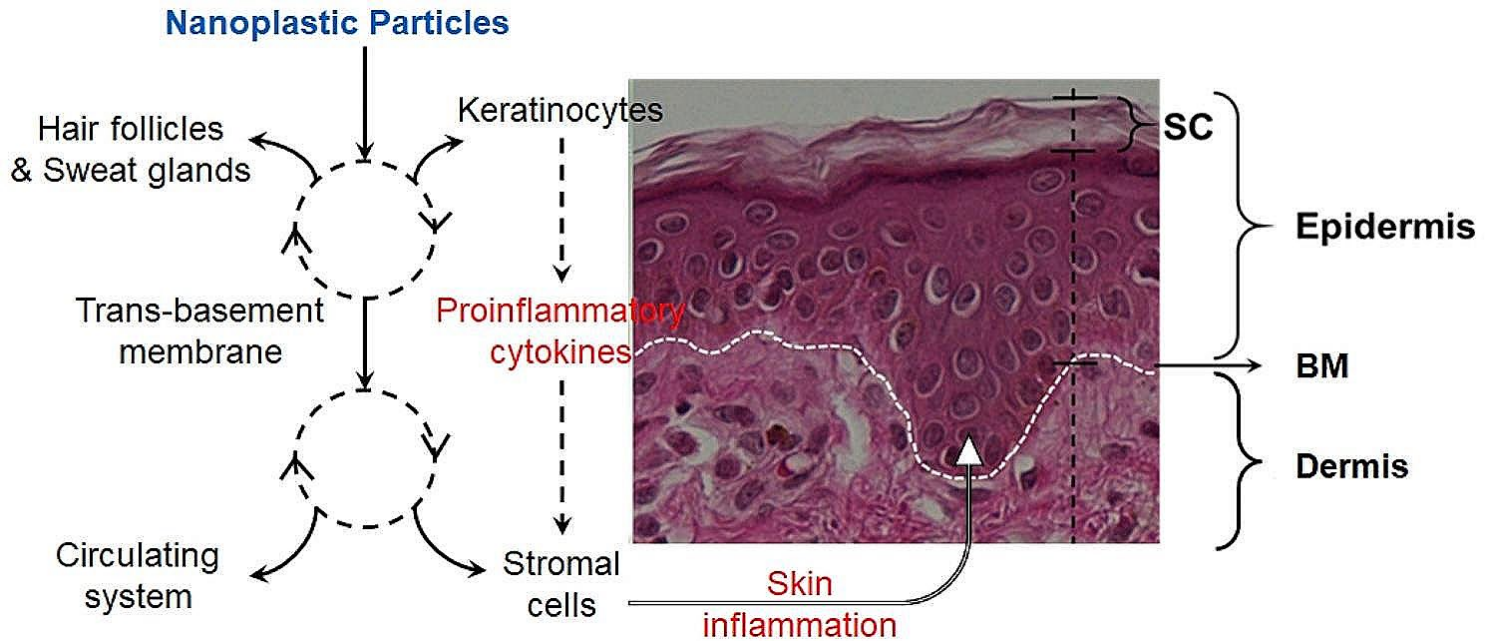
The hypothetical pathways of NP transepidermal penetration (**a**) and genetic response of keratinocytes to NPs (**b**)

In summary, loss of the keratinized barrier really gives the NPs a great chance to enter and penetrate the skin epidermis (Fig. 6). Intracellular NPs will induce the keratinocytes to produce more inflammatory factors, immune response components, as well as signaling molecules. These factors may alter the status of the keratinocytes in the epidermis and also induce the stromal cells in the dermis. These transepidermal NPs may be taken up by stromal cells like dermal fibroblast cells, macrophages, and endothelial cells. They may eventually enter the circulatory system and be transported to other organs (Fig. 6). A recent study identified microplastic particles from human blood [49] suggesting that the NPs are possible to enter the circulatory system through one of the major entry pathways. Furthermore, the circulating NPs can also penetrate the blood-brain barrier to affect the nervous system as reported by another recent study [50]. Due to the limitation of the experimental models used in this study, we are not able to further detect the transportation of NPs inside the dermis. However, it is clear that protection of human skin is critical to prevent the entry of NPs into the human body through skin absorption.

## Conclusion

The entry of NPs into human skin can systematically affect the integumentary system by manipulating the behaviors of many types of cells. The transcript analysis with selected genes suggested a possible induction of the IL-17 regulatory pathway. Additionally, the increased number of myofibroblast cells suggests that an inflammatory response will be initiated by NP accumulation in skin cells. Therefore, skin protection against NPs for patients with compromised barrier function in skin is recommended.

## Materials and methods

### Nanopolystyrene particle uptake by cells

Fluorescently labeled polystyrene nanoplastic particles (NPs) with a diameter of 100–500 nm were purchased from Sigma-Aldrich (St. Louis, MO). Keratinocyte cell line, HaCaT, and GFP expressing HaCaT cells were gifts from Dr. Seok Jong Hong at Northwestern University, Feinberg School of Medicine. Wildtype HaCaT and GFP-labeled HaCaT cells were used in this study. Dermal fibroblast cells were isolated from human skin tissues purchased from the Cooperative Human Tissue Network (CHTN). The epidermis of the skin was completely removed by treatment of 0.25% dispase (Gibco, Billings, Montana) in PBS overnight at 4 °C. The minced dermal tissues were digested overnight with 0.1% collagenase I (Gibco) in DMEM medium at 37 °C with 5% CO_2_ input. Cells isolated from dermal tissue were cultured in DMEM with 10% FBS and antibiotics (Pen-Strep, 1,000 I.U./mL Penicillin and 1,000 µg/mL Streptomycin).

The GFP-HaCaT were grown in the keratinocyte serum-free media (KSFM) supplemented with recombinant epidermal growth factor (rEGF), bovine pituitary extract (BPE), and Pen-Strep antibiotics. The NPs were added to the cell culture media with a final concentration of 1 ppm. Incubated at 37 °C with 5% CO_2_ input for 16 h, the GFP-HaCaT cells and dermal fibroblast cells were thoroughly washed with sterile PBS solution to remove the extracellular NPs. The cells were then briefly fixed in 4% paraformaldehyde (PFA) and observed under a fluorescent microscope (Olympus CKX53SF Inverted fluorescent microscope) or confocal microscope (Zeiss LSM 980 with Airyscan).

### Identification of intracellular NPs

The intracellular NPs taken up by keratinocytes were confirmed by the cryogenic focused ion beam (cryo-FIB) scope at the Center for Integrated Nanotechnologies (CINT, Los Alamos National Laboratory, NM). The keratinocytes were grown on a carbon-coated Au transmission electron microscopy (TEM) grid (Sigma-Aldrich). Prior to the growth of the cells, the grid was coated in 0.05% poly-L-lysine overnight followed by a thorough wash with sterile PBS. The coated grid was deposited at the bottom of a cell in a 24-well cell culture plate. Keratinocytes were seeded to the well at a concentration of 5,000 cells/well. With an overnight growth in KSFM media, the keratinocytes in the well usually reach 50% confluence. Thereafter, the cells were treated with 1 ppm fluorescent NPs overnight and thoroughly washed with PBS to remove the NPs from the background. The grid with the NPs attached to it was then briefly fixed in 4% PFA and flash frozen (vitrified) in liquid ethane and transferred to a Thermo Fisher Scientific Scios 2 dual beam SEM/FIB equipped with a cryogenic stage cooled to -153 °C using cryogenic transfer equipment from Leica Microsystems (VCM, VCT500). A 10 nm layer of Pt was deposited using a cryogenically cooled sputter coater (ACE600) before loading the sample into the cryo-FIB. A protective Pt layer was then deposited and the cells were milled using the Ga^+^ ion beam at 16 keV/2.5 nA, with cleaning cross sections performed at 16 keV/0.5 nA. Imaging was carried out at a low accelerating voltage of 5 keV/50 pA to reduce charging and increase contrast. Nanoparticles with high contrast were observed within the hydrated (vitrified) cellular background. To further characterize the particle, the exposed face was scanned with X-ray energy dispersive spectroscopy (XEDS) using an EDAX Octane Elite detector.

### Skin epidermal penetration by NPs

The penetration of NPs through the skin epidermis was tested using the ex vivo skin culture model [51]. In brief, the full-thickness skin tissue was purchased from the CHTN. Each skin sample was split into two halves. The keratinized epidermal layer, stratum corneum, was removed from the epidermal surface by tape-stripping as previously described [40, 52]. The other half remained the stratum corneum as was. The skin tissues were grafted with a Goulian skin graft knife to generate partial-thickness skin containing the full epidermal layer and a thin dermal layer (10–20% full dermal layer). The skin grafts were then sutured to the outer surface of the cell strainers. The whole construct was supported by the underneath E-medium described in the previous publication [51]. NPs with a diameter of 100 nm were diluted in E-medium to 1 ppm and applied to the top of the skin culture with or without the stratum corneum for 48 h. The proliferation rates of basal epidermal cells in the skin cultures with or without the stratum corneum were estimated using immunohistochemistry with Ki-67 cell proliferation markers. Hematoxylin was used as counterstaining. The NPs in the different layers underneath the stratum corneum were estimated by measuring the total fluorescent signal intensity divided by the total number of epidermal cells in the image.

### Genetic response of keratinocytes to NP accumulation

HaCaT cells were grown in KSFM medium in 6-well plates to a confluence of 80%. The cells were then treated with 1 ppm NPs with various sizes and fluorescent dyes: 100 nm NPs with red fluorescence (R100n), 500 nm NPs with red fluorescence (R500n), 1000 nm NPs with green fluorescence (G1000n), and 1100 nm NPs with no fluorescent tags (NP1100n). The treatment lasted 4 and 16 h and cells were immediately harvested with Trizol Reagent (Invitrogen). RNAs from all samples were purified and the DNAs were removed by the TURBOTM DNase kit (Invitrogen). The cDNAs were made with the SuperScript IV Reverse Transcriptase kit (Invitrogen) following the manufacturer’s protocol and were used for the quantitative PCR (qPCR) of the selected genes. Those genes were possibly involved in the inflammatory response in keratinocytes and were previously tested in our studies [41, 53, 54]. The primers used in this study were synthesized at the Integrated DNA Technologies (IDT DNA, Coralville, IA) and the sequences of the primers were all listed in the Supplementary Data Table S1. The qPCR was performed using the SYBR Green method on a QuantStudio 3 real-time thermocycler (Applied Biosystems, Waltham, MA). The relative gene expression of each sample was calculated using the ΔΔCT method [55]. Based on the results of qPCR, selected genes were screened in the KEGG database for pathway identification [56].

### Transepidermal transportation of NPs in keratinocyte 3D culture

The transportation of NPs within the epidermis was estimated using a 3D keratinocyte culture system, which simulates the stratified squamous epithelial structure in the skin epidermis. The GFP-HaCaT cells were seeded on cell culture inserts (0.4 μm pores) and stratified with the E-medium for 14 days [40, 57]. Fluorescently labeled 100 nm NPs were diluted with E-medium to 1 ppm and applied to the top of the 3D culture. The 3D culture samples were harvested at 4–16 h post-treatment. The transepidermal migration of NPs was visualized using the z-scan imaging function of the Nikon Eclipse Ti2 (Nikon Inc., Tokyo, Japan) fluorescent microscope equipped with a motorized stage.

### Accumulation of transepithelial NPs in dermal fibroblast cells

Primary dermal fibroblast cells were co-cultured with the 3D keratinocyte cultures after the epidermal layers were fully stratified. Similarly, 1 ppm NPs in DMEM with 100 nm diameter were applied to the surface of 3D keratinocyte cultures. The treatments lasted for 4 and 16 h. Thereafter, the co-cultured dermal fibroblast cells were washed with PBS and fixed by 4% PFA. The accumulation of NPs in dermal fibroblast cells was observed under the Olympus CKX53SF Inverted fluorescent microscope. Immunofluorescence with anti-pro-collagen Ia (anti-proCol Ia) and anti-α smooth muscle actin (anti-aSMA) antibodies was used to quantify the production levels of these two protein markers in dermal fibroblast cells. The quantification of the fluorescent signals was performed in ImageJ [58].

### Statistical analyses

The distributions of qPCR, NP uptake, and protein intensity data were analyzed using the Shapiro–Wilk test [36] and Bartlett’s test [59] for the normality test of the datasets and for the homogeneity of data variance, respectively. All datasets were confirmed to be appropriate for the ANOVA and *t*-tests according to the normality and data variance tests. Multiple comparisons between treatment and control groups were performed using one-way ANOVA and Tukey’s post hoc tests. The *p* values from *t*-tests or adjusted *p* values from ANOVA were calculated to demonstrate the significance of the difference between groups.

### Electronic supplementary material

Below is the link to the electronic supplementary material.

Supplementary Material 1

## Data Availability

The datasets used and/or analyzed during the current study are available from the corresponding author upon reasonable request.

## Abbreviations

NP: Nanoplastics
cryo-EM: Cryogenic electron microscopy
KEGG: Kyoto Encyclopedia of Genes and Genomes
XEDS: X-ray energy dispersive spectroscopy
cryo-FIB: Cryogenic focused ion beam
tnfa: Tumor necrosis factor alpha
cxcl2: Chemokine (C-X-C motif) ligand 2
IL: Interleukin
PTGS2: Prostaglandin-endoperoxide synthase 2
COX-2: Cyclooxygenase-2
MMP-1: Matrix metalloproteinase 1
pro-Col: Pro-Collagen
α-SMA: αsmooth muscle actin
CHTN: Cooperative Human Tissue Network
KSFM: Keratinocyte serum-free media
rEGF: Recombinant epidermal growth factor
BPE: Bovine pituitary extract
PFA: Paraformaldehyde
TEM: Transmission electron microscopy
SEM: Scanning electron microscopy CINT: Center for Integrated Nanotechnologies

## References

[1] Andrady AL (2011). Microplastics in the marine environment. Mar Pollut Bull.

[2] Cozar A, Echevarria F, Gonzalez-Gordillo JI, Irigoien X, Ubeda B, Hernandez-Leon S (2014). Plastic debris in the open ocean. Proc Natl Acad Sci U S A.

[3] Julienne F, Delorme N, Lagarde F (2019). From macroplastics to microplastics: role of water in the fragmentation of polyethylene. Chemosphere.

[4] Hernandez LM, Yousefi N, Tufenkji N. Are There Nanoplastics in Your Personal Care Products? Environmental Science & Technology Letters. 2017;4 7:280-5; 10.1021/acs.estlett.7b00187. https://doi.org/10.1021/acs.estlett.7b00187.

[5] Barnes DKA, Galgani F, Thompson RC, Barlaz M. Accumulation and fragmentation of plastic debris in global environments. Philos T R, Soc B. 2009;364 1526:1985-98; 10.1098/rstb.2008.0205.://WOS:000267281600004. 10.1098/rstb.2008.0205 PMC2873009 19528051

[6] Lehner R, Weder C, Petri-Fink A, Rothen-Rutishauser B (2019). Emergence of Nanoplastic in the Environment and possible impact on Human Health. Environ Sci Technol.

[7] Prata JC, da Costa JP, Lopes I, Duarte AC, Rocha-Santos T. Environmental exposure to microplastics: An overview on possible human health effects. Sci Total Environ. 2020;702; doi: ARTN 13445510.1016/j.scitotenv.2019.134455.://WOS:000500590700004. 10.1016/j.scitotenv.2019.134455 31733547

[8] Rahman A, Sarkar A, Yadav OP, Achari G, Slobodnik J. Potential human health risks due to environmental exposure to nano-and microplastics and knowledge gaps: A scoping review. Sci Total Environ. 2021;757; doi: ARTN 14387210.1016/j.scitotenv.2020.143872.://WOS:000604432900098. 10.1016/j.scitotenv.2020.143872 33310568

[9] Bouwmeester H, Hollman PC, Peters RJ (2015). Potential health impact of environmentally released Micro- and nanoplastics in the human food production chain: experiences from Nanotoxicology. Environ Sci Technol.

[10] Xu M, Halimu G, Zhang Q, Song Y, Fu X, Li Y (2019). Internalization and toxicity: a preliminary study of effects of nanoplastic particles on human lung epithelial cell. Sci Total Environ.

[11] Courtene-Jones W, Quinn B, Gary SF, Mogg AOM, Narayanaswamy BE. Microplastic pollution identified in deep-sea water and ingested by benthic invertebrates in the Rockall Trough, North Atlantic Ocean. Environ Pollut. 2017. 10.1016/j.envpol.2017.08.026. https://www.ncbi.nlm.nih.gov/pubmed/28806692. 231 Pt 1:271– 80. 10.1016/j.envpol.2017.08.026 28806692

[12] Moon S, Martin LMA, Kim S, Zhang Q, Zhang R, Xu W, Luo T (2024). Direct observation and identification of nanoplastics in ocean water. Sci Adv.

[13] Ghadially R, Brown BE, Sequeira-Martin SM, Feingold KR, Elias PM (1995). The aged epidermal permeability barrier. Structural, functional, and lipid biochemical abnormalities in humans and a senescent murine model. J Clin Invest.

[14] Kubo A, Nagao K, Amagai M (2012). Epidermal barrier dysfunction and cutaneous sensitization in atopic diseases. J Clin Invest.

[15] Segre JA (2006). Epidermal barrier formation and recovery in skin disorders. J Clin Invest.

[16] Warnberg Gerdin S, Lie A, Asarnoj A, Borres MP, Lodrup Carlsen KC, Fardig M (2022). Impaired skin barrier and allergic sensitization in early infancy. Allergy.

[17] Palmer CN, Irvine AD, Terron-Kwiatkowski A, Zhao Y, Liao H, Lee SP (2006). Common loss-of-function variants of the epidermal barrier protein filaggrin are a major predisposing factor for atopic dermatitis. Nat Genet.

[18] Andrews SN, Jeong E, Prausnitz MR. Transdermal Delivery of Molecules is Limited by Full Epidermis, Not Just Stratum Corneum. Pharm Res-Dordr. 2013;30 4:1099– 109; 10.1007/s11095-012-0946-7.://WOS:000316223300016. 10.1007/s11095-012-0946-7 PMC3596453 23196771

[19] Hwang J, Choi D, Han S, Jung SY, Choi J, Hong J (2020). Potential toxicity of polystyrene microplastic particles. Sci Rep.

[20] Weber A, Schwiebs A, Solhaug H, Stenvik J, Nilsen AM, Wagner M (2022). Nanoplastics affect the inflammatory cytokine release by primary human monocytes and dendritic cells. Environ Int.

[21] Ali N, Katsouli J, Marczylo EL, Gant TW, Wright S, Serna JBD. The potential impacts of micro-and-nano plastics on various organ systems in humans. Ebiomedicine. 2024;99; doi: ARTN 10490110.1016/j.ebiom.2023.104901.://WOS:001134841600001. 10.1016/j.ebiom.2023.104901 PMC10749881 38061242

[22] Oakford ME, Dixon SV, August S, Pickard C, Ardern-Jones M, Lackie P (2011). Migration of immunocytes across the basement membrane in skin: the role of basement membrane pores. J Invest Dermatol.

[23] Alvarez-Roman R, Naik A, Kalia YN, Guy RH, Fessi H (2004). Skin penetration and distribution of polymeric nanoparticles. J Control Release.

[24] Monteiro-Riviere NA, Wiench K, Landsiedel R, Schulte S, Inman AO, Riviere JE (2011). Safety evaluation of sunscreen formulations containing titanium dioxide and zinc oxide nanoparticles in UVB sunburned skin: an in vitro and in vivo study. Toxicol Sci.

[25] Pridgen EM, Alexis F, Farokhzad OC (2015). Polymeric nanoparticle drug delivery technologies for oral delivery applications. Expert Opin Drug Deliv.

[26] Yong JM, Mantaj J, Cheng Y, Vllasaliu D. Delivery of nanoparticles across the intestinal epithelium via the Transferrin Transport Pathway. Pharmaceutics. 2019;11(7). 10.3390/pharmaceutics11070298. https://www.ncbi.nlm.nih.gov/pubmed/31248025. 10.3390/pharmaceutics11070298 PMC6680486 31248025

[27] Fowler R, Vllasaliu D, Trillo FF, Garnett M, Alexander C, Horsley H (2013). Nanoparticle Transport in epithelial cells: Pathway switching through Bioconjugation. Small.

[28] Barua S, Mitragotri S. Challenges associated with penetration of nanoparticles across cell and tissue barriers: a review of current status and future prospects. Nano Today. 2014;9. 10.1016/j.nantod.2014.04.008. 2:223– 43. https://www.ncbi.nlm.nih.gov/pubmed/25132862. 10.1016/j.nantod.2014.04.008 PMC4129396 25132862

[29] Tracy LE, Minasian RA, Caterson EJ. Extracellular Matrix and Dermal Fibroblast Function in the Healing Wound. Adv Wound Care (New Rochelle). 2016;5 3:119– 36; 10.1089/wound.2014.0561. https://www.ncbi.nlm.nih.gov/pubmed/26989578. 10.1089/wound.2014.0561 PMC4779293 26989578

[30] Xie B, Chen MT, Ding PH, Lei L, Zhang X, Zhu D et al. Induction of dermal fibroblasts into dermal papilla cell-like cells in hydrogel microcapsules for enhanced hair follicle regeneration. Appl Mater Today. 2020;21; doi: ARTN 10080510.1016/j.apmt.2020.100805.://WOS:000599826000001.

[31] Nilforoushzadeh MA, Ahmadi Ashtiani HR, Jaffary F, Jahangiri F, Nikkhah N, Mahmoudbeyk M (2017). Dermal fibroblast cells: Biology and function in skin regeneration. J Skin Stem Cell.

[32] Van Hove L, Hoste E (2022). Activation of fibroblasts in skin Cancer. J Invest Dermatol.

[33] Wei K, Nguyen HN, Brenner MB. Fibroblast pathology in inflammatory diseases. J Clin Invest. 2021;131 20; 10.1172/JCI149538. https://www.ncbi.nlm.nih.gov/pubmed/34651581. 10.1172/JCI149538 PMC8516469 34651581

[34] Davidson S, Coles M, Thomas T, Kollias G, Ludewig B, Turley S et al. Fibroblasts as immune regulators in infection, inflammation and cancer. Nat Rev Immunol. 2021;21 11:704– 17; 10.1038/s41577-021-00540-z.://WOS:000645174200001. 10.1038/s41577-021-00540-z 33911232

[35] Russo B, Brembilla NC, Chizzolini C. Interplay Between Keratinocytes and Fibroblasts: A Systematic Review Providing a New Angle for Understanding Skin Fibrotic Disorders. Frontiers in Immunology. 2020;11; doi: ARTN 64810.3389/fimmu.2020.00648.://WOS:000537273000001. 10.3389/fimmu.2020.00648 PMC7232541 32477322

[36] David FN, Johnson NL (1951). The effect of non-normality on the power function of the F-test in the analysis of variance. Biometrika.

[37] Cooper PO, Haas MR, Noonepalle SKR, Shook BA. Dermal Drivers of Injury-Induced Inflammation: Contribution of Adipocytes and Fibroblasts. Int J Mol Sci. 2021;22 4; 10.3390/ijms22041933. https://www.ncbi.nlm.nih.gov/pubmed/33669239. 10.3390/ijms22041933 PMC7919834 33669239

[38] Wang HQ, Smart RC. Overexpression of protein kinase C-alpha in the epidermis of transgenic mice results in striking alterations in phorbol ester-induced inflammation and COX-2, MIP-2 and TNF-alpha expression but not tumor promotion. J Cell Sci. 1999;112. 10.1242/jcs.112.20.3497. (Pt 20):3497– 506. https://www.ncbi.nlm.nih.gov/pubmed/10504298. 10.1242/jcs.112.20.3497 10504298

[39] Masucci MT, Minopoli M, Carriero MV. Tumor Associated Neutrophils. Their Role in Tumorigenesis, Metastasis, Prognosis and Therapy. Front Oncol. 2019;9; doi: ARTN 114610.3389/fonc.2019.01146.://WOS:000499840300001. 10.3389/fonc.2019.01146 PMC6874146 31799175

[40] Xu W, Hong SJ, Zeitchek M, Cooper G, Jia S, Xie P (2015). Hydration status regulates sodium flux and inflammatory pathways through epithelial sodium channel (ENaC) in the skin. J Invest Dermatol.

[41] Xu W, Jia S, Xie P, Zhong A, Galiano RD, Mustoe TA, Hong SJ (2014). The expression of proinflammatory genes in epidermal keratinocytes is regulated by hydration status. J Invest Dermatol.

[42] Yeom M, Lee H, Shin S, Park D, Jung E (2018). PER, a circadian clock component, mediates the suppression of MMP-1 expression in HaCaT keratinocytes by cAMP. Molecules.

[43] Brandner JM, Zacheja S, Houdek P, Moll I, Lobmann R (2008). Expression of matrix metalloproteinases, cytokines, and connexins in diabetic and nondiabetic human keratinocytes before and after transplantation into an ex vivo wound-healing model. Diabetes Care.

[44] Chae S, Piao MJ, Kang KA, Zhang R, Kim KC, Youn UJ (2011). Inhibition of matrix metalloproteinase-1 induced by oxidative stress in human keratinocytes by mangiferin isolated from Anemarrhena asphodeloides. Biosci Biotechnol Biochem.

[45] Jeon S, Lee DK, Jeong J, Yang SI, Kim JS, Kim J, Cho WS. The reactive oxygen species as pathogenic factors of fragmented microplastics to macrophages. Environmental Pollution. 2021;281; doi: ARTN 11700610.1016/j.envpol.2021.117006.://WOS:000656975700002. 10.1016/j.envpol.2021.117006 33812130

[46] Goodman KE, Hua T, Sang QA (2022). Effects of Polystyrene Microplastics on human kidney and liver cell morphology, Cellular Proliferation, and metabolism. ACS Omega.

[47] Lin S, Zhang H, Wang C, Su XL, Song Y, Wu P (2022). Metabolomics Reveal Nanoplastic-Induced mitochondrial damage in Human Liver and Lung cells. Environ Sci Technol.

[48] Ramsperger AFRM, Narayana VKB, Gross W, Mohanraj J, Thelakkat M, Greiner A et al. Environmental exposure enhances the internalization of microplastic particles into cells. Science Advances. 2020;6 50; doi: ARTN eabd121110.1126/sciadv.abd1211.://WOS:000597410300015. 10.1126/sciadv.abd1211 PMC7725476 33298447

[49] Leslie HA, van Velzen MJM, Brandsma SH, Vethaak AD, Garcia-Vallejo JJ, Lamoree MH (2022). Discovery and quantification of plastic particle pollution in human blood. Environ Int.

[50] Shan S, Zhang Y, Zhao H, Zeng T, Zhao X (2022). Polystyrene nanoplastics penetrate across the blood-brain barrier and induce activation of microglia in the brain of mice. Chemosphere.

[51] Xu W, Hong SJ, Jia S, Zhao Y, Galiano RD, Mustoe TA (2012). Application of a partial-thickness human ex vivo skin culture model in cutaneous wound healing study. Lab Invest.

[52] Zhong A, Xu W, Zhao J, Xie P, Jia S, Sun J (2016). S100A8 and S100A9 are Induced by decreased hydration in the Epidermis and promote fibroblast activation and fibrosis in the Dermis. Am J Pathol.

[53] Xu W, Vebrosky EN, Armbrust KL. Potential risk to human skin cells from exposure to dicloran photodegradation products in water. Environ Int. 2018. 10.1016/j.envint.2018.10.010. https://www.ncbi.nlm.nih.gov/pubmed/30343185. 121 Pt 1:861– 70. 10.1016/j.envint.2018.10.010 30343185

[54] Xu W, Vebrosky EN, Armbrust KL (2020). Potential toxic effects of 4-OH-chlorothalonil and photodegradation product on human skin health. J Hazard Mater.

[55] Livak KJ, Schmittgen TD (2001). Analysis of relative gene expression data using real-time quantitative PCR and the 2(-Delta Delta C(T)) method. Methods.

[56] Kanehisa M, Goto S (2000). KEGG: kyoto encyclopedia of genes and genomes. Nucleic Acids Res.

[57] Xu W, Hong SJ, Zhong A, Xie P, Jia S, Xie Z (2015). Sodium channel Nax is a regulator in epithelial sodium homeostasis. Sci Transl Med.

[58] Schneider CA, Rasband WS, Eliceiri KW (2012). NIH Image to ImageJ: 25 years of image analysis. Nat Methods.

[59] Bartlett MS. Properties of sufficiency and statistical tests. Proc R Soc Lon Ser-A. 1937;160 A901:0268– 82; doi: DOI 10.1098/rspa.1937.0109.://WOS:000200958100009.

