# Supplementary material for: Cellular response of keratinocytes to the entry and accumulation of nanoplastic particles
Source: Part Fibre Toxicol. 2024 Apr 29;21:22. doi: 10.1186/s12989-024-00583-9 (PMC11057139; doi:10.1186/s12989-024-00583-9)
Supplement: Supplementary file 1 — Supplementary Material 1 [file 12989_2024_583_MOESM1_ESM.docx]

**Table S1**. Primers for qPCR

| **Target gene** | **Direction** | **Sequence** |
| --- | --- | --- |
| ***gapdh**** | Forward | 5’-TGT TGC CAT CAA TGA CCC CTT-3’ |
|  | Reverse | 5’-CTC CAC GAC GTA CTC AGC G-3’ |
| ***ccl2*** | Forward | 5’-CAG CCA GAT GCA ATC AAT GCC-3’ |
|  | Reverse | 5’-TGG AAT CCT GAA CCC ACT TCT-3’ |
| ***ccl4*** | Forward | 5’-aag ctc tgc gtg act gtc ct-3’ |
|  | Reverse | 5’-gac ttg ctt gcc tct ttt gg-3’ |
| ***cxcl2*** | Forward | 5’-gca ggg aat tca cct caa ga-3’ |
|  | Reverse | 5’-gga ttt gcc att ttt cag ca-3’ |
| ***il1α*** | Forward | 5’-ATC ATG TAA GCT ATG GCC CAC T-3’ |
|  | Reverse | 5’-CTT CCC GTT GGT TGC TAC TAC-3’ |
| ***il1β*** | Forward | 5’-CTC GCC AGT GAA ATG ATG GCT-3’ |
|  | Reverse | 5’-GTC GGA GAT TCG TAG CTG GAT-3’ |
| ***il6*** | Forward | 5’-AAATTCGGTACATCCTCGACGG-3’ |
|  | Reverse | 5’-GGAAGGTTCAGGTTGTTTTCTGC-3’ |
| ***il8*** | Forward | 5’-TTT TGC CAA GGA GTG CTA AAG A-3’ |
|  | Reverse | 5’-AAC CCT CTG CAC CCA GTT TTC-3’ |
| ***mif*** | Forward | 5’-gtt cct ctc cga gct cac c-3’ |
|  | Reverse | 5’-tgc tgt agg agc ggt tct g-3’ |
| ***mmp1*** | Forward | 5’-AGT GAC TGG GAA ACC AGA TGC TGA-3’ |
|  | Reverse | 5’-GCT CTT GGC AAA TCT GGC CTG TAA-3’ |
| ***mmp3*** | Forward | 5’-AGC AAG GAC CTC GTT TTC ATT-3’ |
|  | Reverse | 5’-GTC AAT CCC TGG AAA GTC TTC A-3’ |
| ***mmp9*** | Forward | 5’-AGA CGG GTA TCC CTT CGA CG-3’ |
|  | Reverse | 5’-AAA CCG AGT TGG AAC CAC GAC-3’ |
| ***ptgs2* (COX-2)** | Forward | 5’-GTG CAA CAC TTG AGT GGC TAT-3’ |
|  | Reverse | 5’-AGC AAT TTG CCT GGT GAA TGA T-3’ |
| ***s100a8*** | Forward | 5’-atg ccg tct aca ggg atg ac-3’ |
|  | Reverse | 5’-acg ccc atc ttt atc acc ag-3’ |
| ***s100a9*** | Forward | 5’-GGT CAT AGA ACA CAT CAT GGA GG-3’ |
|  | Reverse | 5’-GGC CTG GCT TAT GGT GGT G-3’ |
| ***s100a12*** | Forward | 5’-CCA ATA CTC AGT TCG GAA GGG G-3’ |
|  | Reverse | 5’-GCA ATG GCT ACC AGG GAT ATG AA-3’ |
| ***tlr4*** | Forward | 5’-ttg gga caa cca gcc taa ag-3’ |
|  | Reverse | 5’-tgc cat tga aag caa ctc tg-3’ |
| ***tnfα*** | Forward | 5’-ATG AGC ACT GAA AGC ATG ATC C-3’ |
|  | Reverse | 5’-GAG GGC TGA TTA GAG AGA GGT C-3’ |

*The house-keeping gene used as an internal reference


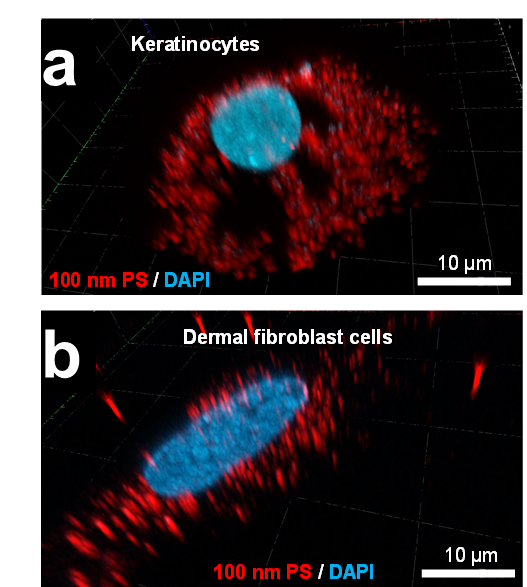


**Fig. S1**. Confocal imaging of Z-sacan of the full-thickness of a keratinocyte (a) and a dermal fibroblast cell (b). Both cells were treated with 100 nm NPs prior to imaging.


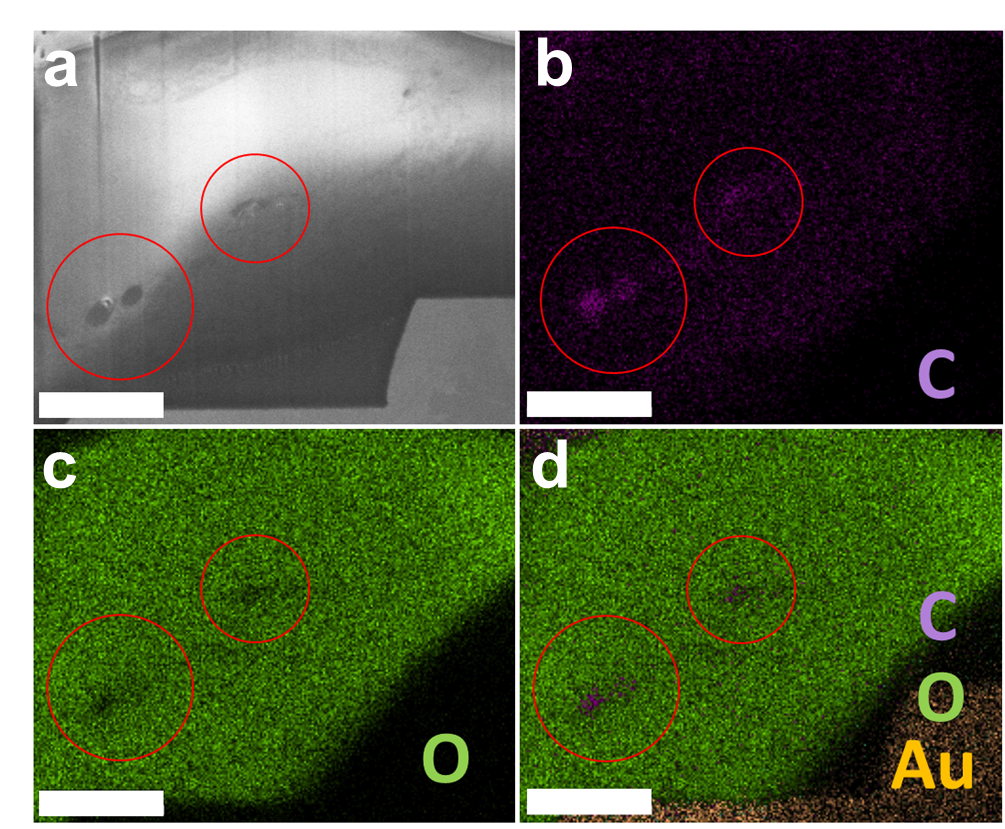


**Fig S2**. Cryo-FIB imaging showed intracellular particles that were possibly polystyrene NPs (a). XEDS mapping demonstrated the strong C signal and weak O signal of the high contrast particles indicate polystyrene (b), as opposed to the weak C signal and strong O signal characteristic of the hydrated cytoplasm (c). Overlapping the two images with the Au background on the grid demonstrated the identified NPs in the cytoplasm (d). Scale bars: 5 µm.


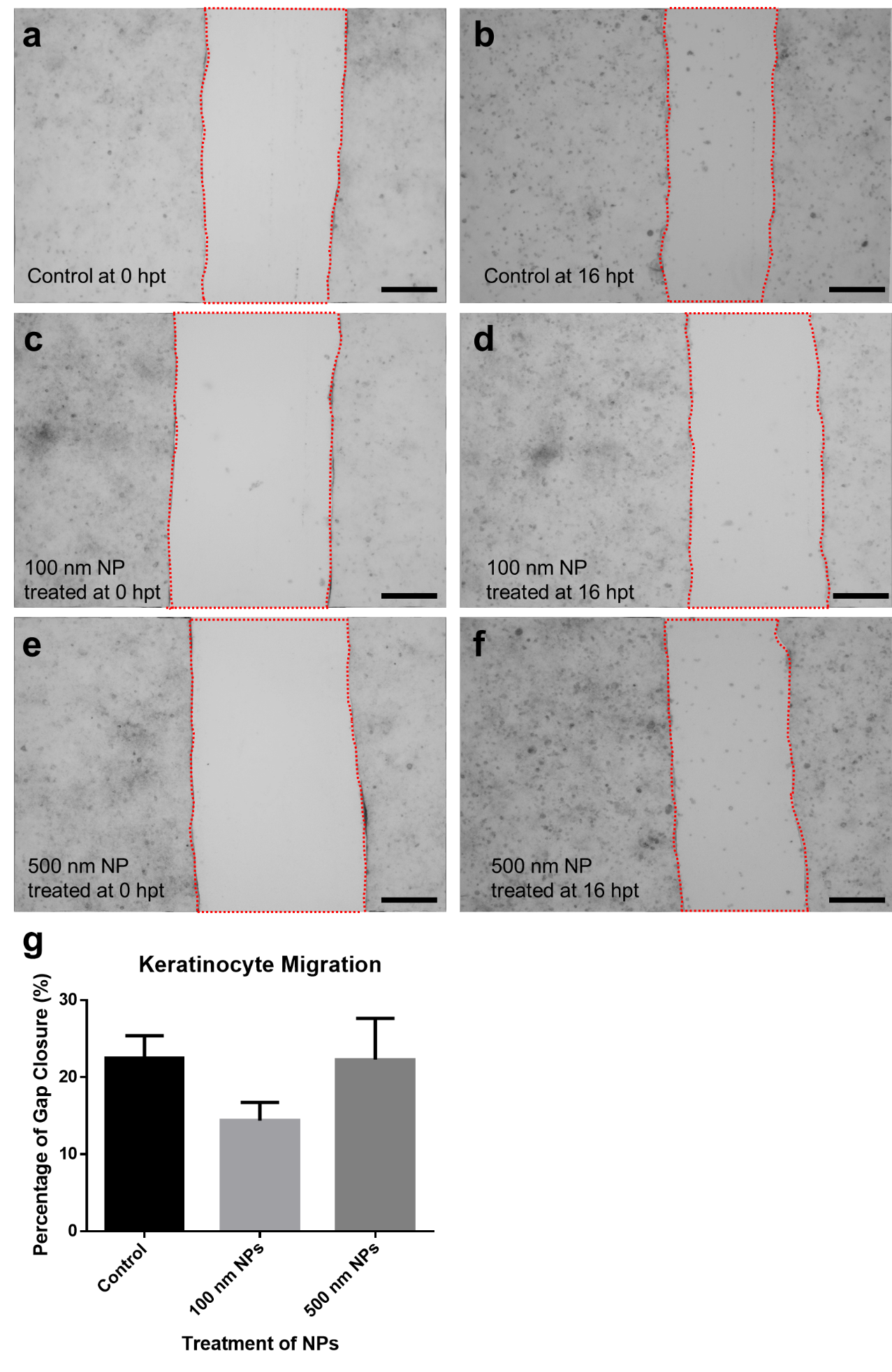


**Fig. S3**. Keratinocytes were grown to about 80% confluence in DMEM supplemented with 10% FBS in 12 well plates. Cells were treated with 1 ppm of 100 nm or 500 nm fluorescent NPs for 24 hours. Cells without the treatment of NPs were used as the control. NP containing media was removed and the cells were thoroughly washed with PBS. The keratinocyte sheet in each well were scratched down the middle with a 200 uL pipette tip (VWR pipet tips, Cat# 37001-598). Each well was then rinsed with DPBS to remove floating cell debris, and DMEM++ media was replaced with DMEM lacking FBS to prevent the accumulation of soluble proteins on NP surfaces. Three images of ach well were taken at 0 and 16 hours to obtain an average gap size (a-f). Gaps were measured by tracing the gaps with the freehand tool in Fiji and dividing the gap area by the average length of each side of the wound. Images were taken using brightfield microscopy at 4x magnification. Percentages of gap closure were calculated as:

$$Gap closure \%=\frac{G\left( 0 \right)-G(16h)}{G\left( 0 \right)}$$

Where the G(0) and G(16h) represented the areas of the gaps at 0 and 16 hours post scratch, respectively (g).


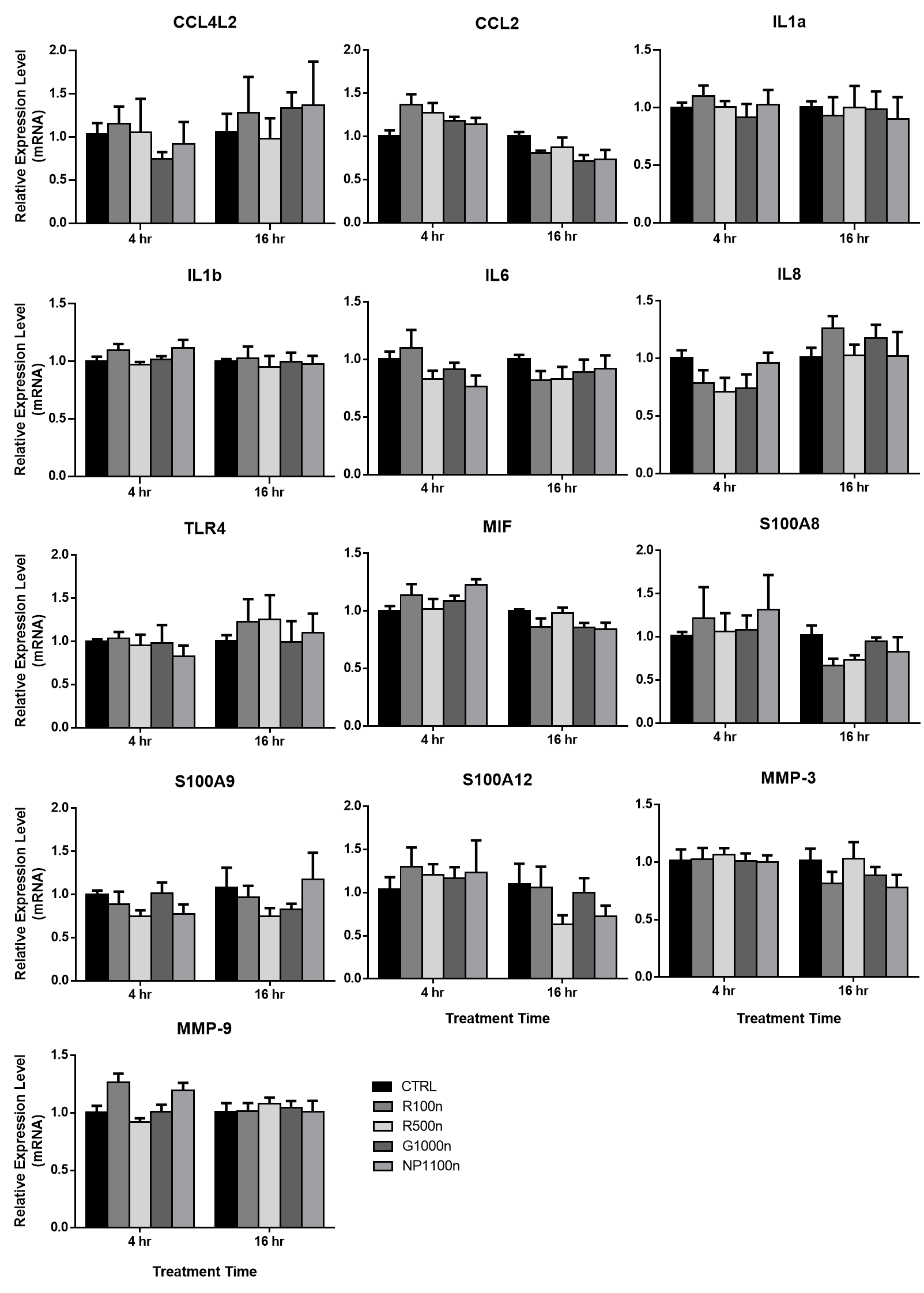


**Fig. S4**. mRNA levels of selected genes in keratinocytes under treatments of control and NPs with different sizes and surface characteristics. CTRL: control without NP treatment; R100n: 100 nm NP with red fluorescence; R500n: 500 nm NP with red fluorescence; G1000n: 1000 nm NP with green fluorescence; NP1100n: 1100 nm NP with no fluorescence. The concentrations of NPs applied to keratinocytes were all 1 ppm.


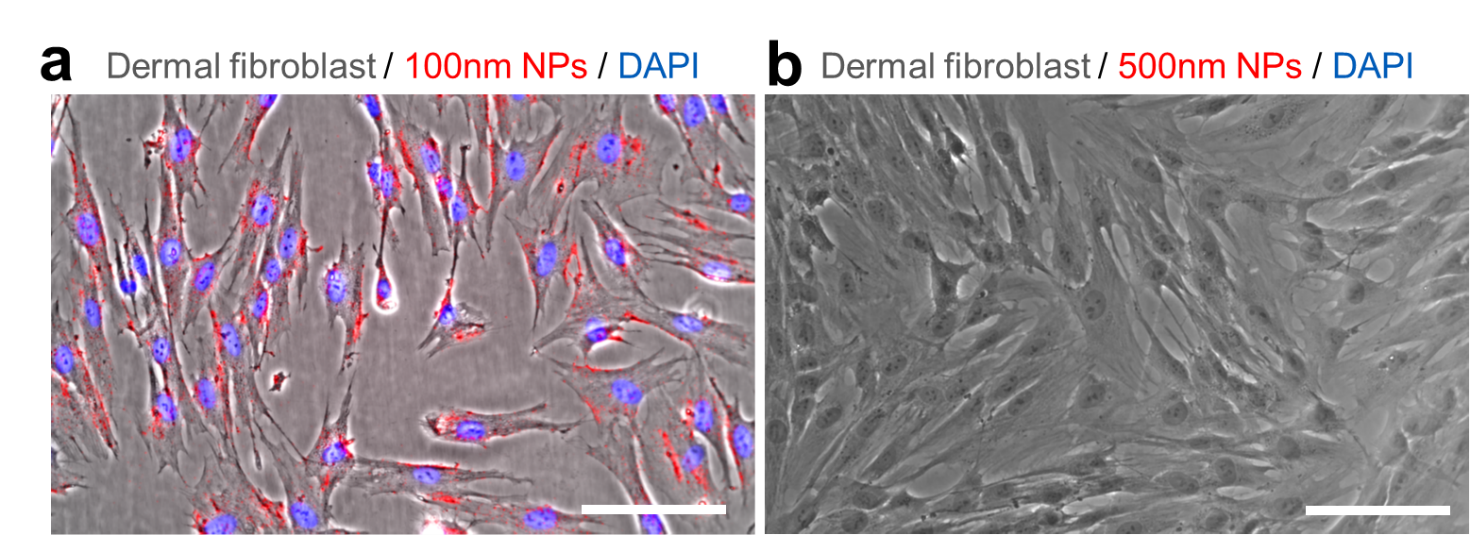


**Fig. S5**. Uptake of NPs passing through the cell culture inserts (0.4 µm pore size) by dermal fibroblast cells. The dermal fibroblast cells were cultured in the wells with the cell culture insert hanging over. No cells were grown on the inserts. Cell culture media containing 1 ppm 100 nm (a) or 500 nm (b) NPs were applied to the inserts. The plates were incubated at 37 °C with 5% CO_2_ input for 24 hours. The uptakes of NPs by the dermal fibroblast cells were observed under fluorescent microscope. Scale bar: 50 µm.
